# Lower Bounds on Multivariate Higher Order Derivatives of Differential Entropy †

**DOI:** 10.3390/e24081155

**Published:** 2022-08-19

**Authors:** Laigang Guo, Chun-Ming Yuan, Xiao-Shan Gao

**Affiliations:** 1 Laboratory of Mathematics and Complex Systems, Ministry of Education, School of Mathematical Sciences, Beijing Normal University, Beijing 100875, China; 2 KLMM, Academy of Mathematics and Systems Science, Chinese Academy of Sciences, Beijing 100190, China; 3 University of Chinese Academy of Sciences, Beijing 100049, China

**Keywords:** differential entropy, completely monotone, Mckean’s conjecture, log-concavity, Gaussian optimality

## Abstract

This paper studies the properties of the derivatives of differential entropy $H(X_{t})$ in Costa’s entropy power inequality. For real-valued random variables, Cheng and Geng conjectured that for m≥1, ${\left(-1\right)}^{m+1}\left(d^{m}/dt^{m}\right)H\left(X_{t}\right)\geq 0$, while McKean conjectured a stronger statement, whereby ${\left(-1\right)}^{m+1}\left(d^{m}/dt^{m}\right)H\left(X_{t}\right)\geq {\left(-1\right)}^{m+1}\left(d^{m}/dt^{m}\right)H\left(X_{Gt}\right)$. Here, we study the higher dimensional analogues of these conjectures. In particular, we study the veracity of the following two statements: $C_{1}\left(m,n\right):{\left(-1\right)}^{m+1}\left(d^{m}/dt^{m}\right)H\left(X_{t}\right)\geq 0$, where *n* denotes that $X_{t}$ is a random vector taking values in $R^{n}$, and similarly, $C_{2}\left(m,n\right):{\left(-1\right)}^{m+1}\left(d^{m}/dt^{m}\right)H\left(X_{t}\right)\geq {\left(-1\right)}^{m+1}\left(d^{m}/dt^{m}\right)H\left(X_{Gt}\right)\geq 0$. In this paper, we prove some new multivariate cases: $C_{1}\left(3,i\right),i=2,3,4$. Motivated by our results, we further propose a weaker version of McKean’s conjecture $C_{3}\left(m,n\right):{\left(-1\right)}^{m+1}\left(d^{m}/dt^{m}\right)H\left(X_{t}\right)\geq {\left(-1\right)}^{m+1}\frac{1}{n}\left(d^{m}/dt^{m}\right)H\left(X_{Gt}\right)$, which is implied by $C_{2}\left(m,n\right)$ and implies $C_{1}\left(m,n\right)$. We prove some multivariate cases of this conjecture under the log-concave condition: $C_{3}\left(3,i\right),i=2,3,4$ and $C_{3}\left(4,2\right)$. A systematic procedure to prove $C_{l}\left(m,n\right)$ is proposed based on symbolic computation and semidefinite programming, and all the new results mentioned above are explicitly and strictly proved using this procedure.

## 1. Introduction

Shannon’s entropy power inequality (EPI) is one of the most important information inequalities [1], which has many proofs, generalizations, and applications [2,3,4,5,6,7,8,9,10,11]. In particular, Costa presented a generalized version of the EPI in his seminal paper [12].

Let *X* be an n-dimensional random vector with finite variance and a probability density function p(x). For t>0, define $X_{t}≜X+Z_{t}$, where $Z_{t}∼N_{n}\left(0,tI\right)$ is an independent standard Gaussian random vector with the covariance matrix t×I. The *probability density* of $X_{t}$ is

(1) $$p_{t}\left(x_{t}\right)=\frac{1}{{\left(2\pi t\right)}^{n/2}}\int_{R^{n}}p\left(x\right)\exp\left(-\frac{∥x_{t}{-x∥}^{2}}{2t}\right)dx.$$

Thus, the heat equation holds for $p_{t}\left(x_{t}\right)$, i.e.,

(2) $$\frac{dp_{t}}{dt}=\frac{1}{2}\nabla^{2}p_{t}.$$

*The differential entropy* of $X_{t}$ is defined as

(3) $$H\left(X_{t}\right)=-\int_{R^{n}}p_{t}\left(x_{t}\right)\log p_{t}\left(x_{t}\right)dx_{t}.$$

Costa [12] proved that the *entropy power* of $X_{t}$, given by $N\left(X_{t}\right)=\frac{1}{2\pi e}e^{\left(2/n\right)H\left(X_{t}\right)}$ is a concave function in *t*. More precisely, Costa proved $\left(d/dt\right)N\left(X_{t}\right)\geq 0$ and $\left(d^{2}/dt^{2}\right)N\left(X_{t}\right)\leq 0$.

Due to its importance, several new proofs and generalizations for Costa’s EPI have been given. Dembo [13] gave a simple proof for Costa’s EPI via the Fisher information inequality. Villani [14] proved Costa’s EPI with Cauchy–Schwarz inequality as well as the heat equation. Toscani [15] proved that $\left(d^{3}/dt^{3}\right)N\left(X_{t}\right)\geq 0$ if $p_{t}$ is log-concave. Cheng and Geng proposed a conjecture [16]:

**Conjecture** **1.** *The first derivative of $H(X_{t})$ (i.e., the Fisher information) is*completely monotone *in t, that is,*

(4) $$C_{1}\left(m,n\right):\;\;{\left(-1\right)}^{m+1}\left(d^{m}/dt^{m}\right)H\left(X_{t}\right)\geq 0.$$

*Costa’s EPI implies $C_{1}\left(1,n\right)$ and $C_{1}\left(2,n\right)$ [12], and Cheng–Geng proved $C_{1}\left(3,1\right)$ and $C_{1}\left(4,1\right)$ [16].*

Let $X_{G}∼N_{n}\left(\mu ,\sigma^{2}I\right)$ be an *n*-dimensional Gaussian random vector and $X_{Gt}≜X_{G}+Z_{t}$ be the Gaussian $X_{t}$. McKean [17] proved that $X_{Gt}$ achieves the minimum of $\left(d/dt\right)H\left(X_{t}\right)$ and $-(d^{2}/dt^{2})$$H(X_{t})$ is subject to Var$\left(X_{t}\right)=\sigma^{2}+t$, and conjectured the general case:

**Conjecture** **2.** *The following inequality holds subject to Var$\left(X_{t}\right)=\sigma^{2}+t$,*

(5) $$\begin{array}{c} C_{2}\left(m,n\right):\;\;{\left(-1\right)}^{m+1}\left(d^{m}/dt^{m}\right)H\left(X_{t}\right)\geq {\left(-1\right)}^{m+1}\left(d^{m}/dt^{m}\right)H\left(X_{Gt}\right)\geq 0. \end{array}$$

McKean proved $C_{2}\left(1,1\right)$ and $C_{2}\left(2,1\right)$[17]. Zhang–Anantharam–Geng [18] proved $C_{2}\left(3,1\right)$, $C_{2}\left(4,1\right)$ and $C_{2}\left(5,1\right)$ if the probability density function of $X_{t}$ is log-concave. Note that $C_{2}\left(1,n\right)$ and $C_{2}\left(2,n\right)$ are immediate consequences of Entropy Power Inequality and Costa’s concavity of entropy power result [12], respectively. In this paper, we notice that in the multivariate case, Conjecture 2 might not be true for m>2 even under the log-concave condition, which motivates us to propose the following weaker conjecture:

**Conjecture** **3.** *The following inequality holds subject to Var$\left(X_{t}\right)=\sigma^{2}+t$,*

(6) $$\begin{array}{c} C_{3}\left(m,n\right):\;\;{\left(-1\right)}^{m+1}\left(d^{m}/dt^{m}\right)H\left(X_{t}\right)\geq {\left(-1\right)}^{m+1}\frac{1}{n}\left(d^{m}/dt^{m}\right)H\left(X_{Gt}\right)\geq 0. \end{array}$$

We see that Conjecture 3 coincides with Conjecture 2 for n=1 (univariate case). Additionally, Conjecture 2 implies Conjecture 3 and Conjecture 3 implies Conjecture 1. The three conjectures give different lower bounds for the derivatives of ${\left(-1\right)}^{m+1}H\left(X_{t}\right)$.

**Remark** **1.** *The authors in [14,16] proved some cases of Conjecture 1 by writing the left-hand formula in Conjecture 1 as sums of squares and, hence, concluded their sign. We provide a systematic way to explore this idea using symbolic computation and semidefinite programming and prove several new results in the multivariate cases.*

Our procedure for proving $C_{s}\left(m,n\right)$ consists of three main ingredients. First, a systematic method is proposed to compute the constraints $R_{i},i=1,\ldots ,N_{1}$ that are satisfied by $p_{t}\left(x_{t}\right)$ and its derivatives. The condition that $p_{t}$ is log-concave can also be reduced to a set of constraints, i.e., $R_{j},j=1,\ldots ,N_{2}$. Second, based on symbolic computation, proof for $C_{s}\left(m,n\right)$ is reduced to the following problem:

(7) $$\exists p_{i}\in R\;\mathrm{and}\;Q_{j}\;s.t.\;\left(E-\sum_{i=1}^{N_{1}}p_{i}R_{i}-\sum_{j=1}^{N_{2}}Q_{j}R_{j}=S\right)$$

where $E,Q_{j},$ and *S* are polynomials in $p_{t}$ and its derivatives such that *E* represents the conjecture, $Q_{j}\geq 0$, and *S* is a sum of squares (SOS). Third, problem (7) can be solved with semidefinite programming (SDP) [19,20]. Note that from Equation (7), we can give an explicit and strict proof for $C_{s}\left(m,n\right)$.

Using the procedure proposed in this paper, we prove several new results about the three conjectures: $C_{1}\left(3,2\right)$, $C_{1}\left(3,3\right)$, $C_{1}\left(3,4\right)$, and $C_{3}\left(3,2\right)$, $C_{3}\left(3,3\right)$, $C_{3}\left(3,4\right)$, $C_{3}\left(4,2\right)$ under the log-concave condition.

In Table 1, we give the data for computing the SOS representation (7) using the Matlab software in Appendix A of [21], where Vars is the number of variables, and $N_{1}$ and $N_{2}$ are the numbers of constraints in (7).

The procedure is inspired by the work of [12,14,16,18], and uses basic ideas introduced therein. The specific contributions in this paper are:

(1) Based on symbolic computation and semidefinite programming, $C_{s}\left(m,n\right)$ can be automatically verified with the aid of the software systems Maple and Matlab, and analytical proofs for $C_{s}\left(m,n\right)$ can also be efficiently produced.
(2) The new concept of differentially homogenous polynomials is introduced and used to reduce the computational complexity. Compared with the pure SDP-based approach (such as [18]), the computational efficiency of our procedure is, in general, much higher. See Procedure 2 for details.
(3) The results in [16,18] are generalized from the univariate cases to the multivariate cases (new results). This is the first attempt for the multivariate high order cases of the conjectures.
(4) In comparison to the literature (such as [12,15,16,18]), the constraints (integral or log-concave) considered in this paper are more general.

The rest of this paper is organized as follows. In Section 2, we give the proof procedure. In Section 3, we prove $C_{1}\left(3,2\right)$, $C_{1}\left(3,3\right)$ and $C_{1}\left(3,4\right)$. In Section 4 we prove $C_{3}\left(3,2\right)$, $C_{3}\left(3,3\right)$, and $C_{3}\left(3,4\right)$ under the log-concave condition. In Section 5, we prove $C_{3}\left(4,2\right)$ under the log-concave condition. In Section 6, the conclusions are presented.

## 2. Proof Procedure

In this section, we provide a general procedure to prove $C_{s}\left(m,n\right)$ for specific values of s,m, and *n*.

### 2.1. Some Notations

Let ${\left[n\right]}_{0}=\left\{0,1,\ldots ,n\right\}$, [n]={1,…,n}, and $x_{t}=\left[x_{1,t},\ldots ,x_{n,t}\right]$. To simplify the notations, we use $p_{t}$ to denote $p_{t}\left(x_{t}\right)$ in the rest of the paper. Denote

$$P_{n}=\left\{\frac{\partial^{h}p_{t}}{\partial^{h_{1}}x_{1,t}\cdots \partial^{h_{n}}x_{n,t}}:h=\sum_{i=1}^{n}h_{i},h_{i}\in N\right\}$$

to be the set of all derivatives of $p_{t}$ with respect to the differential operators $\frac{\partial }{\partial x_{i,t}},i=1,\ldots ,n$ and $R[P_{n}]$ to be the set of polynomials in $P_{n}$ with coefficients in R. For $v\in P_{n}$, let ord(v) be the order of *v*. For a monomial $\prod_{i=1}^{r}v_{i}^{d_{i}}$ with $v_{i}\in P_{n}$, its *degree*, *order*, and *total order* are defined as $\sum_{i=1}^{r}d_{i}$, $\max_{i=1}^{r}\mathrm{ord}\left(v_{i}\right)$, and $\sum_{i=1}^{r}d_{i}\cdot \mathrm{ord}\left(v_{i}\right)$, respectively.

A polynomial in $R[P_{n}]$ is called a *k*th-order *differentially homogeneous polynomial* or simply a *k*th-order *differential form*, if all its monomials have a degree of *k* and a total order of *k*. Let $M_{k,n}$ be the set of all monomials which have a degree of *k* and a total order of *k*. Then, the set of *k*th-order differential forms is an R-linear vector space generated by $M_{k,n}$, which is denoted as ${\mathrm{Span}}_{R}\left(M_{k,n}\right)$.

We will use Gaussian elimination in ${\mathrm{Span}}_{R}\left(M_{k,n}\right)$ by treating the monomials as variables. We always use the *lexicographic order for the monomials* to be defined below unless mentioned otherwise. Consider two distinct derivatives $v_{1}=\frac{\partial^{h}p_{t}}{\partial^{h_{1}}x_{1,t}\cdots \partial^{h_{n}}x_{n,t}}$ and $v_{2}=\frac{\partial^{s}p_{t}}{\partial^{s_{1}}x_{1,t}\cdots \partial^{s_{n}}x_{n,t}}$. We say $v_{1}>v_{2}$ if h>s, or h=s, $h_{l}>s_{l}$ and $h_{j}=s_{j}$ for j=l+1,…,n. Consider the two distinct monomials $m_{1}=\prod_{i=1}^{r}v_{i}^{d_{i}}$ and $m_{2}=\prod_{i=1}^{r}v_{i}^{e_{i}}$, where $v_{i}\in P_{n}$ and $v_{i}<v_{j}$ for i<j. We define $m_{1}>m_{2}$ if $d_{l}>e_{l}$, and $d_{i}=e_{i}$ for i=l+1,…,r.

From (1), $p_{t}:R^{n+1}\to R$ is a function in $x_{t}$ and *t*. Therefore, each polynomial $f\in R[P_{n}]$ is also a function in $x_{t}$ and *t*, $\tilde{f}\left(t\right)=\int_{R^{n}}fdx_{t}$ is a function in *t*, and the *expectation* of *f* with respect to $x_{t}$ $E\left[f\right]≜\int_{R^{n}}p_{t}fdx_{t}$ is also a function in *t*. By f≥0, $\tilde{f}\geq 0$, and E[f]≥0, we mean $f(x_{t},t)\geq 0$, $\tilde{f}\left(t\right)\geq 0$, and E[f](t)≥0 for all $x_{t}\in R^{n}$ and t>0.

### 2.2. Three Parts of the Proof

In this section, we give the procedure to prove $C_{s}\left(m,n\right)$, which consists of three parts.

#### 2.2.1. Part I

In **step 1**, we reduce the proof of $C_{s}\left(m,n\right)$ into the proof of an integral inequality, as shown by the following lemma, whose proof will be given in Section 2.3:

**Lemma** **1.** *Proof that $C_{s}\left(m,n\right),s=1,2,3$ can be reduced to show*

(8) $$\begin{array}{c} \int_{R^{n}}\frac{E_{s,m,n}}{p_{t}^{2m-1}}dx_{t}\geq 0 \end{array}$$

*where*

$$\begin{array}{c} E_{s,m,n}=\sum_{a_{1}=1}^{n}\cdots \sum_{a_{m}=1}^{n}E_{s,m,n,a_{m}},\\ a_{m}=\left(a_{1},\ldots ,a_{m}\right), \end{array}$$

*$E_{s,m,n,a_{m}}$ is a 2mth-order differential form in $R[P_{m,n}]$, and*

(9) $$\begin{array}{c} P_{m,n}=\left\{\frac{\partial^{h}p_{t}}{\partial^{h_{1}}x_{a_{1},t}\cdots \partial^{h_{m}}x_{a_{m},t}}:h\in {\left[2m-1\right]}_{0};a_{i}\in \left[n\right],i\in \left[m\right]\right\}. \end{array}$$

#### 2.2.2. Part II

In **step 2**, we compute the constraints which are relations satisfied by the probability density $p_{t}$ of $X_{t}$. In this paper, we consider two types of constraints: integral constraints and log-concave constraints, which will be given in Lemmas 2 and 3, respectively. Since $E_{s,m,n}$ in (8) is a 2mth-order differential form, we need only the constraints which are 2mth-order differential forms.

**Definition** **1.** *An mth-order*integral constraint *is the 2mth-order differential form R in $R[P_{n}]$ such that*

$$\int_{R^{n}}\;\frac{R}{p_{t}^{2m-1}}dx_{t}=0.$$

**Lemma** **2** ([22]). *There is a systematic method to compute the mth-order integral constraints $C_{m,n}=\left\{R_{i},i=1,\ldots ,N_{1}\right\}$.*

A function $f:R^{n}\to R$ is called *log-concave* if logf is a concave function. In this paper, by the *log-concave condition*, we mean that the density function $p_{t}$ is log-concave.

**Definition** **2.** *An mth-order*log-concave constraint *is a 2mth-order differential form R in $R[P_{n}]$ such that R≥0 under the log-concave condition.*

The following lemma computes the log-concave constraints:

**Lemma** **3** ([22]). *Let $H\left(p_{t}\right)\in R{\left[P_{n}\right]}^{n\times n}$ be the Hessian matrix of $p_{t}$, $\nabla p_{t}=\left(\frac{\partial p_{t}}{\partial x_{1,t}},\ldots ,\frac{\partial p_{t}}{\partial x_{n,t}}\right)$,*

(10) $$L\left(p_{t}\right)≜p_{t}H\left(p_{t}\right)-\nabla^{T}p_{t}\nabla p_{t},$$

*and ${▵}_{k,l},l=1,\ldots ,L_{k}$ be the kth-order principle minors of $L(p_{t})$. Then, the mth-order log-concave constraints are*

(11) $$C_{m,n}=\left\{\prod_{i=1}^{l}{\left(-1\right)}^{k_{i}}{▵}_{k_{i},l_{i}}T_{k_{1},\ldots ,k_{l}}\;|\;\sum_{i=1}^{l}k_{i}\leq m\right\}$$

*where $T_{k_{1},\ldots ,k_{l}}\in {\mathrm{Span}}_{R}\left(M_{2m-2\sum_{i=1}^{l}k_{i},n}\right)$ and $T_{k_{1},\ldots ,k_{l}}\geq 0$.*

Note that $T_{k_{1},\ldots ,k_{l}}$ in (11) are not known. For convenience, denote

(12) $$C_{m,n}=\left\{P_{j},j=1,\ldots ,N_{2}\right\},$$

where $P_{j}$ represents $\prod_{i=1}^{l}{\left(-1\right)}^{k_{i}}{▵}_{k_{i},l_{i}}$ in (11). From Lemma 3, it is easy to see that $\prod_{i=1}^{l}{\left(-1\right)}^{k_{i}}$ ${▵}_{k_{i},l_{i}}$ is a $\left(2\sum_{i=1}^{l}k_{i}\right)$th-order log-concave constraint.

#### 2.2.3. Part III

In **step 3**, we give a procedure to write $E_{s,m,n}$ as an SOS under the constraints, the details of which will be given in Section 2.4.

**Procedure** **1.** *For $E_{s,m,n}$ in Lemma 1, $C_{m,n}=\left\{R_{i},i=1,\ldots ,N_{1}\right\}$ in Lemma 2, and $C_{m,n}=\left\{P_{j},j=1,\ldots ,N_{2}\right\}$ in Lemma 3, the procedure computes $e_{l}\in R$ and $Q_{j}\in {\mathrm{Span}}_{R}\left(M_{2m-\deg P_{j},n}\right)$ such that*

(13) $$\begin{array}{ccc} & & E_{s,m,n}-\sum_{i=1}^{N_{1}}e_{i}R_{i}-\sum_{j=1}^{N_{2}}P_{j}Q_{j}=S, \end{array}$$

(14) $$\begin{array}{ccc} & & \mathrm{and}\;Q_{j}\geq 0,j=1,\ldots ,N_{2}, \end{array}$$

*where S is an SOS. If the log-concave condition is not needed, we may set $Q_{j}=0$ for all j.*

To summarize the proof procedure, we have the following:

**Theorem** **1.** *If Procedure 1 satisfies (13) and (14) for certain s,m, and n, then $C_{s}\left(m,n\right)$ is explicitly and strictly proved.*

**Proof.**  With Lemma 1, we have the following proof for $C_{s}\left(m,n\right)$:

(15) $$\begin{array}{cc} \int_{R}\frac{E_{t,m,n}}{p_{t}^{2m-1}}dx_{t}\;\;\;\;\; & \overset{(13)}{=}\int_{R}\frac{\sum_{i=1}^{N_{1}}e_{i}R_{i}+\sum_{j=1}^{N_{2}}P_{j}Q_{j}+S}{p_{t}^{2m-1}}dx_{t}\\ \; & \overset{S1}{=}\int_{R}\frac{\sum_{j=1}^{N_{2}}P_{j}Q_{j}+S}{p_{t}^{2m-1}}dx_{t}\\ \; & \overset{S2}{\geq }\int_{R}\frac{S}{p_{t}^{2m-1}}dx_{t}\\ \; & \overset{S3}{\geq }0. \end{array}$$

Equality S1 is true, because $R_{i}$ is an integral constraint by Lemma 2. By Lemma 3 and (14), $P_{j}Q_{j}\geq 0$ is true under the log-concave condition, so inequality S2 is true under the log-concave condition. Finally, inequality S3 is true, because S≥0 is an SOS. □

### 2.3. Proof of Lemma 1

Costa [12] proved the following basic properties for $p_{t}$ and $H(X_{t})$,

(16) $$\begin{array}{cc} \frac{dH(X_{t})}{dt}\;\;\;\; & =-\frac{1}{2}E\left[\nabla^{2}\log p_{t}\right]\\ \; & =\frac{1}{2}\int_{R^{n}}\frac{∥\nabla p_{t}{∥}^{2}}{p_{t}}dx_{t}\\ \; & =\frac{1}{2}J\left(X_{t}\right), \end{array}$$

where

$$\begin{array}{c} \nabla p_{t}=\left(\frac{\partial p_{t}}{\partial x_{1,t}},\ldots ,\frac{\partial p_{t}}{\partial x_{n,t}}\right),\;\nabla^{2}p_{t}=\sum_{i=1}^{n}\frac{\partial^{2}p_{t}}{\partial^{2}x_{i,t}}, \end{array}$$

and $J\left(X_{t}\right)≜E\left(\frac{∥\nabla p_{t}{∥}^{2}}{p_{t}^{2}}\right)$ is the *Fisher information* [6]. Equation (16) implies $C_{1}\left(1,n\right)$: $\frac{d}{dt}H\left(X_{t}\right)\geq 0$.

For s=1, Lemma 1 was proved by

**Lemma** **4** ([22]). *For $m\in N_{m>1}$, we have*

(17) $$\begin{array}{c} {\left(-1\right)}^{m+1}\left(d^{m}/dt^{m}\right)H\left(X_{t}\right)=\int_{R^{n}}\frac{E_{1,m,n}}{p_{t}^{2m-1}\left(x_{t}\right)}dx_{t}, \end{array}$$

*where*

$$\begin{array}{cc} E_{1,m,n}\;\;\; & =\frac{{\left(-1\right)}^{m+1}p_{t}^{2m-1}}{2}\frac{d^{m-1}}{dt^{m-1}}\left(\frac{∥\nabla p_{t}{∥}^{2}}{p_{t}}\right)\\ & =\sum_{a_{1}=1}^{n}\cdots \sum_{a_{m}=1}^{n}E_{1,m,n,a_{m}} \end{array}$$

*is a 2mth-order differential form in $R[P_{m,n}]$.*

To prove Lemma 1 for s=2,3, we need to compute $\left(d^{m}/dt^{m}\right)H\left(X_{Gt}\right)$. Let $X_{G}∼N_{n}\left(\mu ,\sigma^{2}I\right)$ be an *n*-dimensional Gaussian random vector and $X_{Gt}≜X_{G}+Z_{t}$, where $Z_{t}∼N_{n}\left(0,tI\right)$ is introduced in Section 1. Then, $X_{Gt}∼N_{n}\left(\mu ,\left(\sigma^{2}+t\right)I\right)$ and the probability density of $X_{Gt}$ is

$$\begin{array}{c} {\hat{p}}_{t}=\frac{1}{{\left(2\pi \left(\sigma^{2}+t\right)\right)}^{n/2}}\;\exp(-\frac{1}{2(\sigma^{2}+t)}∥x_{t}-\mu {∥}^{2}). \end{array}$$

**Lemma** **5** ([22]). *Let $T=\nabla^{2}\log p_{t}$ and $T_{G}=\nabla^{2}\log{\hat{p}}_{t}$. Then, under the log-concave condition, we have*

(18) $$\begin{array}{c} E\left[{\left(-T\right)}^{m}\right]\overset{\left(a\right)}{\geq }{\left[E\left(-T\right)\right]}^{m}\overset{\left(b\right)}{\geq }{\left[E\left(-T_{G}\right)\right]}^{m}\\ \overset{\left(c\right)}{=}{\left(-1\right)}^{m+1}\frac{2n^{m-1}}{(m-1)!}\left(d^{m}/dt^{m}\right)H\left(X_{Gt}\right). \end{array}$$

**Lemma** **6** ([22]). *For $T=\nabla^{2}\log p_{t}$ and $m\in N_{m>1}$, we have*

(19) $$E\left[{\left(-T\right)}^{m}\right]=\int_{R}^{n}\frac{E_{0,m,n}}{p_{t}^{2m-1}}dx_{t}$$

*where*

$$\begin{array}{c} E_{0,m,n}=\sum_{a_{1}=1}^{n}\cdots \sum_{a_{m}=1}^{n}E_{0,m,n,a_{m}},\\ a_{m}=\left(a_{1},\ldots ,a_{m}\right), \end{array}$$

*and $E_{0,m,n,a_{m}}$ is a 2mth-order differential form in $R[P_{m,n}]$.*

We can now prove Lemma 1 for s=2,3. Let

(20) $$\begin{array}{c} E_{2,m,n}=E_{1,m,n}-\frac{(m-1)!}{2n^{m-1}}E_{0,m,n},\\ E_{3,m,n}=E_{1,m,n}-\frac{(m-1)!}{2n^{m}}E_{0,m,n}, \end{array}$$

where $E_{1,m,n}$ and $E_{0,m,n}$ are from Lemmas 4 and 6, respectively. By Lemma 5, $C_{s}\left(m,n\right)$ is true if $\int_{R^{n}}\frac{E_{s,m,n}}{p_{t}^{2m-1}}dx_{t}\geq 0$ for l=2,3. Together with Lemma 4, Lemma 1 is proved.

### 2.4. Main Result (Procedure 1)

In this section, we present the detailed Procedure 1, called Procedure 2, which is based on symbolic computation and the SOS theory.

**Procedure 2. Input** *: $E_{s,m,n}$ and $R_{i},i=1,\ldots ,N_{1}$ are 2mth-order differential forms in $R[P_{n}]$; $P_{j},j=1,\ldots ,N_{2}$ are $2k_{j}$th-order differential forms in in $R[P_{n}]$.*

**Output** *: $e_{i}\in R$ and $Q_{j}\in {\mathrm{Span}}_{R}\left(M_{2(m-k_{j}),n}\right)$ such that (13) and (14) are true, or fail meaning such that $e_{i}$ and $Q_{j}$ are not found.*

**S1**. Treat the monomials in $M_{m,n}$ as new variables $m_{l},l=1,\ldots ,N_{m,n}$, which are all the monomials in $R[P_{n}]$ with the degree *m* and the total order *m*. We call $m_{l}m_{s}$ a *quadratic monomial*.

**S2**. Write monomials in $C_{m,n}=\left\{R_{i},i=1,\ldots ,N_{1}\right\}$ as quadratic monomials if possible. By performing Gaussian elimination on $C_{m,n}$ by treating the monomials as variables and according to a monomial order such that a quadratic monomial is less than a non-quadratic monomial, we obtain

$${\tilde{C}}_{m,n}=C_{m,n,1}\cup C_{m,n,2},$$

where $C_{m,n,1}$ is the set of quadratic forms in $m_{i}$, $C_{m,n,2}$ is the set of non-quadratic forms, and ${\mathrm{Span}}_{R}\left(C_{m,n}\right)={\mathrm{Span}}_{R}\left({\tilde{C}}_{m,n}\right)$.

**S3**. There may exist relationships among the variables $m_{i}$, which are called *intrinsic constraints*. For instance, for $m_{1}=p_{t}^{2}{\left(\frac{\partial^{2}p_{t}}{\partial^{2}x_{1,t}}\right)}^{2}$, $m_{2}=p_{t}{\left(\frac{\partial p_{t}}{\partial x_{1,t}}\right)}^{2}\frac{\partial^{2}p_{t}}{\partial^{2}x_{1,t}}$, and $m_{3}={\left(\frac{\partial p_{t}}{\partial x_{1,t}}\right)}^{4}$ in $M_{4,n}$, an intrinsic constraint is $m_{1}m_{3}-m_{2}^{2}=0$. By adding the intrinsic constraints which are quadratic forms in $m_{i}$ to $C_{m,n,1}$, we obtain

$${\hat{C}}_{m,n,1}=\left\{{\hat{R}}_{i},i=1,\ldots ,N_{3}\right\}.$$

**S4**. Let $M_{2(m-k_{j}),n}=\left\{m_{j,k},k=1,\ldots ,V_{j}\right\}$ and $Q_{j}=\sum_{k=1}^{V_{j}}q_{j,k}m_{j,k}$, where $q_{j,k}$ are variables to be found later. Let ${\bar{R}}_{j}$ be obtained from $P_{j}Q_{j}$ by writing monomials in $P_{j}Q_{j}$ as quadratic monomials in $m_{i}$, and eliminating the non-quadratic monomials with $C_{m,n,2}$, such that ${\bar{R}}_{j}-P_{j}Q_{j}\in {\mathrm{Span}}_{R}\left(C_{m,n}\right)$ and ${\bar{R}}_{j}=\sum_{l=1}^{V_{j}}q_{j,l}h_{j,l}$, where $h_{j,l}\in R\left[m_{i},P_{n}\right]$. If an $h_{j,l}$ is not a quadratic form in $m_{i}$, then delete ${\bar{R}}_{j}$; hence, the ${\bar{R}}_{j}$’s in quadratic form are selected. Then, denote these constraints as $R_{j},j=1,\ldots ,N_{2}$, which form the reduced set ${\hat{C}}_{m,n}$.

**S5**. Let ${\hat{E}}_{s,m,n}$ be obtained from $E_{s,m,n}$ by eliminating the non-quadratic monomials using $C_{m,n,2}$ such that $E_{s,m,n}-{\hat{E}}_{s,m,n}\in {\mathrm{Span}}_{R}\left(C_{m,n,2}\right)\subset {\mathrm{Span}}_{R}\left(C_{m,n}\right)$.

**S6**. Since ${\hat{E}}_{s,m,n}$, ${\hat{R}}_{i},i=1,\ldots ,N_{3}$ and $R_{j},j=1,\ldots ,N_{2}$ are quadratic forms in $m_{i}$, we can use the Matlab codes given in Appendix A [21] to compute $p_{i},q_{j,s}\in R$ such that

(21) $$\begin{array}{cc} & {\hat{E}}_{s,m,n}-\sum_{i=1}^{N_{3}}p_{i}{\hat{R}}_{i}-\sum_{j=1}^{N_{2}}R_{j}=S,\\ & \;\;R_{j}=\sum_{l=1}^{V_{j}}q_{j,l}h_{j,l},j=1,\ldots ,N_{2} \end{array}$$

(22) $$\begin{array}{cc} & Q_{j}=\sum_{l=1}^{V_{j}}q_{j,l}m_{j,l}\geq 0,j=1,\ldots ,N_{2} \end{array}$$

where

$$S=\sum_{i=1}^{N_{m,n}}c_{i}{\left(\sum_{j=i}^{N_{m,n}}e_{ij}m_{j}\right)}^{2}$$

is an SOS, $c_{i},e_{ij}\in R$ and $c_{i}\geq 0$. If (21) and (22) cannot be found, return FAIL.

**S7**. Since ${\hat{R}}_{i}$, $E_{s,m,n}-{\hat{E}}_{s,m,n}$, $R_{j}-P_{j}Q_{j}$ are all in ${\mathrm{Span}}_{R}\left(C_{m,n}\right)$, Equations (13) and (14) can be obtained from (21) and (22), respectively.

**Remark** **2.** *Procedure 2 can be implemented automatically by Maple and Matlab on a computer. In Procedure 2, steps* ***S2*** *,* ***S4*** *and* ***S5*** *are based on the symbolic computation theory for reduction, which makes our method more efficient than the pure SDP-based method [18] or a direct theoretical proof [16]. The use of symbolic computation also ensures that our calculation is strict and free of numerical errors.*

**Remark** **3.** *Let R be an intrinsic constraint. Then, R becomes zero when replacing $m_{i}$ by its corresponding monomial in $M_{m,n}$. Therefore, ${\mathrm{Span}}_{R}\left({\hat{C}}_{m,n,1}\right)={\mathrm{Span}}_{R}\left(C_{m,n,1}\right)\subset {\mathrm{Span}}_{R}\left(C_{m,n}\right)$ in $R[P_{n}]$; that is, we do not need to include the intrinsic constraints in (21). However, these intrinsic constraints are needed when using the Matlab software in Appendix A of [21].*

### 2.5. An Illustrative Example

As an illustrative example, we prove $C_{2}\left(3,1\right)$ under the log-concave condition using the proof procedure given in Section 2.2. Since n=1, denote

$$x_{t}=x_{1,t},f:=f_{0}:=p_{t},f_{n}:=\frac{\partial^{n}p_{t}}{\partial^{n}x_{1,t}},\;n\in N_{>0}.$$

In **step 1**, by Lemma 1 and (8), we have

(23) $$\begin{array}{c} \frac{d^{3}H\left(X_{t}\right)}{dt^{3}}-\frac{2!}{2}E\left[\frac{{\left(f_{1}^{2}-ff_{2}\right)}^{3}}{f^{6}}\right]\\ \overset{(16)}{=}\int \left(\frac{1}{2}\frac{d^{2}}{dt^{2}}\left(\frac{f_{1}^{2}}{f}\right)-\frac{{\left(f_{1}^{2}-ff_{2}\right)}^{3}}{f^{5}}\right)dx_{t}\\ \overset{(8)}{=}\int \frac{E_{2,3,1}}{f^{5}}dx_{t} \end{array}$$

where

$$\begin{array}{cc} E_{2,3,1}\;\;\; & =\frac{1}{4}f^{4}f_{3}^{2}-\frac{1}{2}f^{3}f_{1}f_{3}f_{2}+\frac{1}{4}f^{4}f_{1}f_{5}-\frac{11}{4}f^{2}f_{1}^{2}f_{2}^{2}\\ & -\frac{1}{8}f^{3}f_{1}^{2}f_{4}+f^{3}f_{2}^{3}+3ff_{1}^{4}f_{2}-f_{1}^{6} \end{array}$$

is a sixth-order differential form.

In **step 2**, we compute the constraints with Lemmas 2 and 3. With Lemma 2, we find six third-order integral constraints: $C_{3,1}=\left\{R_{i},i=1,\ldots ,6\right\}$:

$$\begin{array}{c} R_{1}=5ff_{1}^{4}f_{2}-4f_{1}^{6},\\ R_{2}=2f^{3}f_{1}f_{2}f_{3}+f^{3}f_{2}^{3}-2f^{2}f_{1}^{2}f_{2}^{2},\\ R_{3}=f^{4}f_{1}f_{5}+f^{4}f_{2}f_{4}-f^{3}f_{1}^{2}f_{4},\\ R_{4}=f^{3}f_{1}^{2}f_{4}+2f^{3}f_{1}f_{2}f_{3}-2f^{2}f_{1}^{3}f_{3},\\ R_{5}=f^{2}f_{1}^{3}f_{3}+3f^{2}f_{1}^{2}f_{2}^{2}-3ff_{1}^{4}f_{2},\\ R_{6}=f^{4}f_{2}f_{4}+f^{4}f_{3}^{2}-f^{3}f_{1}f_{2}f_{3}. \end{array}$$

With Lemma 3, we obtain one third-order log-concave constraint: $C_{3,1}=\left\{P_{1}Q_{1}\right\}$, where

$$P_{1}=ff_{2}-f_{1}^{2},\;Q_{1}\in {\mathrm{Span}}_{R}\left(M_{4,1}\right),\;\mathrm{and}\;Q_{1}\geq 0.$$

In **step 3**, we use Procedure 2 to compute the SOS representation (13) and (14) with the input $E_{2,3,1},C_{3,1}=\left\{R_{i},i=1,\ldots ,6\right\},P_{1}=f_{1}^{2}-ff_{2}$.

**S1**. The new variables are $M_{3,1}=\left\{m_{1}=f^{2}f_{3},m_{2}=ff_{1}f_{2},m_{3}=f_{1}^{3}\right\}$, which are listed from high to low in the lexicographical monomial order.

**S2**. By writing monomials in $C_{3,1}$ as quadratic monomials in $m_{i}$ if possible and performing Gaussian elimination on $C_{3,1}$, we have

$$\begin{array}{cc} C_{3,1,1}=\{\; & \;\;\;\;\;\;{\hat{R}}_{1}=5m_{2}m_{3}-4m_{3}^{2},\\ \; & \;\;\;\;\;\;{\hat{R}}_{2}=m_{1}m_{3}+3m_{2}^{2}-\frac{12}{5}m_{3}^{2}\},\\ C_{3,1,2}=\{\; & \;\;\;\;\;\;{\tilde{R}}_{1}=f^{3}f_{2}^{3}+2m_{1}m_{2}-2m_{2}^{2},\\ \; & \;\;\;\;\;\;{\tilde{R}}_{2}=f^{4}f_{1}f_{5}-m_{1}^{2}+3m_{1}m_{2}+6m_{2}^{2}-\frac{24}{5}m_{3}^{2},\\ \; & \;\;\;\;\;\;{\tilde{R}}_{3}=f^{4}f_{2}f_{4}+m_{1}^{2}-m_{1}m_{2},\\ \; & \;\;\;\;\;\;{\tilde{R}}_{4}=f^{3}f_{1}^{2}f_{4}+2m_{1}m_{2}+6m_{2}^{2}-\frac{24}{5}m_{3}^{2}\}. \end{array}$$

**S3**. There exist no intrinsic constraints and thus, ${\hat{C}}_{3,1,1}=\left\{{\hat{R}}_{1},{\hat{R}}_{2}\right\}$ and $N_{3}=2$.

**S4**. $M_{4,1}=\left\{f^{3}f_{4},f^{2}f_{1}f_{3},f^{2}f_{2}^{2},ff_{1}^{2}f_{2},f_{1}^{4}\right\}$. Then, $Q_{1}=q_{1,1}f^{2}f_{2}^{2}+q_{1,2}ff_{1}^{2}f_{2}+q_{1,3}f_{1}^{4}$.

Monomials $f^{3}f_{4},f^{2}f_{1}f_{3}$ do not appear in $Q_{1}$ due to $Q_{1}\geq 0$. By writing monomials in $P_{1}Q_{1}$ as quadratic monomials if possible and using $C_{3,1,2}$ to eliminate non-quadratic monomials, we obtain

$$\begin{array}{cc} R_{1}\;\;\; & =P_{1}Q_{1}-\left(\frac{1}{5}q_{1,2}{\hat{R}}_{1}-q_{1,1}{\tilde{R}}_{1}-\frac{1}{5}q_{1,3}{\hat{R}}_{1}\right)\\ & =q_{1,1}\left(2m_{1}m_{2}-m_{2}^{2}\right)+q_{1,2}\left(\frac{4}{5}m_{3}^{2}-m_{2}^{2}\right)+\frac{q_{1,3}}{5}m_{3}^{2}. \end{array}$$

**S5**. By writing $E_{2,3,1}$ as a quadratic form in $m_{i}$, we have

$$\begin{array}{cc} {\hat{E}}_{2,3,1}\;\;\; & =E_{2,3,1}-\frac{3}{5}{\hat{R}}_{1}-{\tilde{R}}_{1}-\frac{1}{4}{\tilde{R}}_{2}+\frac{1}{8}{\tilde{R}}_{4}\\ & =\frac{1}{2}m_{1}^{2}-3m_{1}m_{2}-\frac{3}{2}m_{2}^{2}+2m_{3}^{2}. \end{array}$$

**S6**. Since ${\hat{E}}_{3,1}$, ${\hat{R}}_{1}$, ${\hat{R}}_{2}$, $R_{1}$ are quadratic forms in $m_{i}$, we can use the Matlab software in Appendix A of [21] to obtain the following SOS representation

(24) $$\begin{array}{c} {\hat{E}}_{2,3,1}=\sum_{i=1}^{2}p_{i}{\hat{R}}_{i}+R_{1}+\sum_{i=1}^{3}c_{i}{\left(\sum_{j=i}^{3}e_{i,j}m_{j}\right)}^{2},\\ Q_{1}\geq 0, \end{array}$$

where

$$\begin{array}{c} p_{1}=\frac{6}{5},\;p_{2}=-2,c_{1}=\frac{1}{2},e_{1,1}=1,e_{1,2}=-3,e_{1,3}=2,\\ q_{1,1}=q_{1,2}=q_{1,3}=c_{2}=c_{3}=0. \end{array}$$

**S7**. We obtain

$$\begin{array}{cc} E_{2,3,1}\;\;\;\; & =\frac{3}{4}R_{1}+R_{2}+\frac{1}{4}R_{3}+\frac{1}{8}R_{4}-\frac{7}{4}R_{5}-\frac{1}{4}R_{6}\\ & +\sum_{i=1}^{3}c_{i}{\left(\sum_{j=i}^{3}e_{i,j}m_{j}\right)}^{2}. \end{array}$$

From Theorem 1 and (23), we have

(25) $$\begin{array}{cc} \; & \frac{d^{3}H\left(X_{t}\right)}{dt^{3}}-\frac{2!}{2}E\left[\frac{{\left(f_{1}^{2}-ff_{2}\right)}^{3}}{f^{6}}\right]\\ \; & =\int_{R}\frac{E_{2,3,1}}{p_{t}^{5}}dx_{t}\\ \; & \overset{}{=}\int_{R}\frac{1}{p_{t}^{5}}(\frac{3}{4}R_{1}+R_{2}+\frac{1}{4}R_{3}+\frac{1}{8}R_{4}\\ \; & -\frac{7}{4}R_{5}-\frac{1}{4}R_{6}+\sum_{i=1}^{3}c_{i}{\left(\sum_{j=i}^{3}e_{i,j}m_{j}\right)}^{2})\;dx_{t}\\ \; & \overset{}{=}\int_{R}\frac{{\left(m_{1}-3m_{2}+2m_{3}\right)}^{2}}{2p_{t}^{5}}dx_{t}\\ \; & \overset{}{\geq }0. \end{array}$$

Thus, an explicit and strict proof is given for $C_{2}\left(3,1\right)$. Note that this example is also considered in [18] by the pure SDP-based method, which is a semi-automatic algorithm. See Table 1 for the time used to provide analytical proof of this example by our automatic method on a computer.

## 3. Proof of C_1_(3,n) for n = 2, 3, 4

In this section, we use the procedure in Section 2.2 to prove $C_{1}\left(3,n\right)$ for n=2,3,4.

### 3.1. Compute E_1,3,n_

In **step 1**, we compute $E_{1,3,n}$ in (8) and (20):

(26) $$\frac{1}{2}\frac{d^{2}}{dt^{2}}\left(\int_{R^{n}}\frac{∥\nabla p_{t}{∥}^{2}}{p_{t}}dx_{t}\right)\overset{\left(2\right)}{=}\int_{R^{n}}\frac{E_{1,3,n}}{p_{t}^{5}}dx_{t},$$

where

$$E_{1,3,n}=\sum_{a=1}^{n}\sum_{b=1}^{n}\sum_{c=1}^{n}F_{3,a,b,c}$$

and

$$\begin{array}{cc} F_{3,a,b,c}\;\;\;\;\; & =\frac{p_{t}^{4}}{4}\frac{\partial^{3}p_{t}}{\partial x_{a,t}\partial^{2}x_{c,t}}\frac{\partial^{3}p_{t}}{\partial x_{a,t}\partial^{2}x_{b,t}}-\frac{p_{t}^{3}}{4}\frac{\partial p_{t}}{\partial x_{a,t}}\frac{\partial^{3}p_{t}}{\partial x_{a,t}\partial^{2}x_{b,t}}\frac{\partial^{2}p_{t}}{\partial^{2}x_{c,t}}\\ \; & +\frac{p_{t}^{4}}{4}\frac{\partial p_{t}}{\partial x_{a,t}}\frac{\partial^{5}p_{t}}{\partial x_{a,t}\partial^{2}x_{b,t}\partial^{2}x_{c,t}}-\frac{p_{t}^{3}}{4}\frac{\partial p_{t}}{\partial x_{a,t}}\frac{\partial^{3}p_{t}}{\partial x_{a,t}\partial^{2}x_{c,t}}\frac{\partial^{2}p_{t}}{\partial^{2}x_{b,t}}\\ \; & +\frac{p_{t}^{2}}{4}{\left(\frac{\partial p_{t}}{\partial x_{a,t}}\right)}^{2}\frac{\partial^{2}p_{t}}{\partial^{2}x_{b,t}}\frac{\partial^{2}p_{t}}{\partial^{2}x_{c,t}}-\frac{p_{t}^{3}}{8}{\left(\frac{\partial p_{t}}{\partial x_{a,t}}\right)}^{2}\frac{\partial^{4}p_{t}}{\partial^{2}x_{b,t}\partial^{2}x_{c,t}}. \end{array}$$

### 3.2. Compute the Third-Order Constraints

In **step 2**, we obtain the third-order constraints. We introduce the notation

(27) $$V_{a,b,c}=\left\{\frac{\partial^{h}p_{t}}{\partial^{h_{1}}x_{a,t}\partial^{h_{2}}x_{b,t}\partial^{h_{3}}x_{c,t}}:h=h_{1}+h_{2}+h_{3}\in {\left[5\right]}_{0}\right\},$$

where a,b,c are variables taking values in [n]. Then,

$$P_{3,n}=\cup_{a=1}^{n}\cup_{b=1}^{n}\cup_{c=1}^{n}V_{a,b,c}.$$

The third-order integral constraints are:

(28) $$C_{3,n}=\left\{R_{i,a,b,c}^{\left(3\right)},\;:\;i=1,\ldots ,955;a,b,c\in \left[n\right]\right\},$$

where $R_{i,a,b,c}^{\left(3\right)}$ in the form of lengthy formulas can be found in [23]. Note that we do not use all the third-order constraints in [23].

### 3.3. Proof of C_1_(3,2)

The proof follows Procedure 2 with $E_{1,3,2}$ given in (26) as the input. To make the proof explicit, we will give the key expressions.

In Step **S1**, the new variables are $M_{3,2}$ and are listed in the lexicographical monomial order:

$$\begin{array}{c} m_{1}=p_{t}^{2}\frac{\partial p_{t}^{3}}{\partial^{3}x_{2,t}},\;m_{2}=p_{t}^{2}\frac{\partial^{3}p_{t}}{\partial x_{1,t}\partial^{2}x_{2,t}},\\ m_{3}=p_{t}^{2}\frac{\partial^{3}p_{t}}{\partial^{2}x_{1,t}\partial x_{2,t}},\;m_{4}=p_{t}^{2}\frac{\partial p_{t}^{3}}{\partial^{3}x_{1,t}},\\ m_{5}=p_{t}\frac{\partial^{2}p_{t}}{\partial^{2}x_{2,t}}\frac{\partial p_{t}}{\partial x_{2,t}},\;m_{6}=p_{t}\frac{\partial^{2}p_{t}}{\partial^{2}x_{2,t}}\frac{\partial p_{t}}{\partial x_{1,t}},\\ m_{7}=p_{t}\frac{\partial^{2}p_{t}}{\partial x_{1,t}\partial x_{2,t}}\frac{\partial p_{t}}{\partial x_{2,t}},\;m_{8}=p_{t}\frac{\partial^{2}p_{t}}{\partial x_{1,t}\partial x_{2,t}}\frac{\partial p_{t}}{\partial x_{1,t}},\\ m_{9}=p_{t}\frac{\partial^{2}p_{t}}{\partial x_{1,t}^{2}}\frac{\partial p_{t}}{\partial x_{2,t}},\;m_{10}=p_{t}\frac{\partial^{2}p_{t}}{\partial x_{1,t}^{2}}\frac{\partial p_{t}}{\partial x_{1,t}},\\ m_{11}={\left(\frac{\partial p_{t}}{\partial x_{2,t}}\right)}^{3},\;m_{12}={\left(\frac{\partial p_{t}}{\partial x_{2,t}}\right)}^{2}\frac{\partial p_{t}}{\partial x_{1,t}},\\ m_{13}=\frac{\partial p_{t}}{\partial x_{2,t}}{\left(\frac{\partial p_{t}}{\partial x_{1,t}}\right)}^{2},\;m_{14}={\left(\frac{\partial p_{t}}{\partial x_{1,t}}\right)}^{3}. \end{array}$$

In Step **S2**, the constraints are

$$C_{3,2}=\left\{R_{j,a,b,c}^{\left(3\right)}\;:\;j=1,\ldots ,955;a,b,c\in \left[2\right]\right\}.$$

Removing the repeated ones, we have $N_{1}=135$. We obtain $C_{3,2,1}$ and $C_{3,2,2}$, which contain 48 and 52 constraints, respectively.

In Step **S3**, there exist 15 intrinsic constraints:

$$\begin{array}{c} m_{5}m_{8}=m_{6}m_{7},\;m_{5}m_{10}=m_{6}m_{9},\;m_{5}m_{12}=m_{6}m_{11},\\ m_{5}m_{13}=m_{6}m_{12},\;m_{5}m_{14}=m_{6}m_{13},\;m_{7}m_{10}=m_{8}m_{9},\\ m_{7}m_{12}=m_{8}m_{11},\;m_{7}m_{13}=m_{8}m_{12},\;m_{7}m_{14}=m_{8}m_{13},\\ m_{9}m_{12}=m_{10}m_{11},\;m_{9}m_{13}=m_{10}m_{12},\;m_{9}m_{14}=m_{10}m_{13},\\ m_{11}m_{13}=m_{12}^{2},\;m_{11}m_{14}=m_{12}m_{13},\;m_{12}m_{14}=m_{13}^{2}. \end{array}$$

Thus, ${\hat{C}}_{3,2,1}$ contains 63 constraints and $N_{3}=63$.

Step **S4** is not needed in the proof of this case.

In Step **S5**, by eliminating the non-quadratic monomials in $E_{1,3,2}$ using $C_{3,2,2}$ to obtain a quadratic form in $m_{i}$ and then simplifying the quadratic form using $C_{3,2,1}$, we have

$$\begin{array}{cc} {\hat{E}}_{1,3,2}\;\;\;\;\; & =E_{1,3,2}-(\frac{3}{4}{\hat{R}}_{17}-\frac{1}{6}{\hat{R}}_{12}-\frac{1}{6}{\hat{R}}_{13}+\frac{7}{6}{\hat{R}}_{18}-\frac{1}{2}{\hat{R}}_{32}\\ \; & -\frac{1}{2}{\hat{R}}_{34}-\frac{5}{8}{\hat{R}}_{35}-\frac{1}{2}{\hat{R}}_{40}-\frac{1}{12}{\tilde{R}}_{2}-\frac{1}{8}{\tilde{R}}_{5}-\frac{1}{4}{\tilde{R}}_{6}\\ \; & +\frac{1}{2}{\tilde{R}}_{7}+\frac{1}{4}{\tilde{R}}_{8}+\frac{1}{2}{\tilde{R}}_{18}+\frac{1}{4}{\tilde{R}}_{19}-\frac{1}{8}{\tilde{R}}_{39}-\frac{1}{4}{\tilde{R}}_{46}\\ \; & +\frac{1}{2}{\tilde{R}}_{48}-\frac{1}{8}{\tilde{R}}_{49}+\frac{1}{4}{\tilde{R}}_{53})\\ \; & =\frac{1}{2}m_{1}^{2}-m_{1}m_{5}+\frac{3}{2}m_{2}^{2}-3m_{2}m_{6}+\frac{3}{2}m_{3}^{2}+\frac{1}{2}m_{4}^{2}\\ \; & -2m_{4}m_{6}-m_{4}m_{7}-m_{4}m_{10}-\frac{1}{2}m_{5}^{2}+\frac{3}{2}m_{6}^{2}-3m_{7}^{2}\\ \; & -2m_{7}m_{10}+3m_{8}^{2}-\frac{5}{2}m_{9}^{2}-\frac{3}{2}m_{9}m_{11}+21m_{9}m_{13}\\ \; & -\frac{1}{2}m_{10}^{2}+\frac{3}{5}m_{11}^{2}+3m_{12}^{2}-15m_{13}^{2}+\frac{3}{5}m_{14}^{2}. \end{array}$$

In Step **S6**, using the Matlab program in [23] with ${\hat{E}}_{1,3,2}$ and ${\hat{C}}_{3,2,1}$ as the input, we find an SOS representation for ${\hat{E}}_{1,3,2}$. Thus, by Theorem 1, $C_{1}\left(3,2\right)$ is strictly proved.

### 3.4. Proof of C_1_(3,3)

The proof follows Procedure 2 with $E_{1,3,3}$ given in (29) as the input. The detailed lengthy formulas can be seen in [23].

In Step **S1**, the new variables are $M_{3,3}=\left\{m_{i},i=1,\ldots ,38\right\}$ which is the set of all monomials in $R[P_{3,3}]$ with a degree of 3 and a total order of 3, and which are listed in the lexicographical monomial order.

In Step **S2**, the constraints are: $C_{3,n}=\left\{R_{i,a,b,c}^{\left(3\right)}\;:\;i=1,\ldots ,955\right\}$, $N_{1}=955$. We obtain $C_{3,n,1}$ and $C_{3,n,2}$, which contain 350 and 328 constraints, respectively.

In Step **S3**, there exist 189 intrinsic constraints. In total, ${\hat{C}}_{3,n,1}$ contains 539 constraints. Using R-Gaussian elimination in ${\mathrm{Span}}_{R}\left({\hat{C}}_{3,n,1}\right)$ shows that 512 of these 539 constraints are linearly independent, so $N_{3}=512$.

Step **S4** is not needed in the proof of this case.

In Step **S5**, by eliminating the non-quadratic monomials in $E_{1,3,3}$ using $C_{3,3,2}$ and then simplifying the expression using $C_{3,3,1}$, we obtain ${\hat{E}}_{1,3,3}$ written as a quadratic form in $m_{i}$.

In Step **S6**, using the Matlab program in [23] with ${\hat{E}}_{1,3,3}$ and ${\hat{C}}_{3,3,1}$ as the input, we find an SOS representation for ${\hat{F}}_{3,3}$. Thus, using Theorem 1, $C_{1}\left(3,3\right)$ is strictly proved.

### 3.5. Proof of C_1_(3,4)

The proof follows Procedure 2 with $E_{1,3,4}$ given in (29) as the input. The detailed lengthy formulas can be seen in [23].

In Step **S1**, the new variables are $M_{3,4}=\left\{m_{i},i=1,\ldots ,80\right\}$ which is the set of all monomials in $R[P_{3,4}]$ with a degree of 3 and a total order of 3, and which are listed in the lexicographical monomial order.

In Step **S2**, we obtain $C_{3,4}=\left\{R_{i,a,b,c}^{\left(3\right)},R_{j}^{\left(0\right)},R_{k,a,b}^{\left(2\right)},\;:\;i=1,\ldots ,955,j=1,\ldots ,8,k=1,\ldots ,20,a,b,c\in \left[4\right]\right\}$. Removing the repeated ones, we have $N_{1}=3172$. We obtain $C_{3,4,1}$ and $C_{3,4,2}$ which contain 1120 and 975 constraints, respectively.

In Step **S3**, there exist 1080 intrinsic constraints. In total, ${\hat{C}}_{3,4,1}$ contains 2200 constraints. Only 1966 constraints in ${\hat{C}}_{3,4,1}$ are R-linearly independent, so $N_{2}=1966$.

Step **S4** is not needed in the proof of this case.

In Step **S5**, by eliminating the non-quadratic monomials in $E_{1,3,4}$ using $C_{3,4,2}$ to obtain a quadratic form in $m_{i}$ and then simplifying the quadratic form with $C_{3,4,1}$, we obtain ${\hat{E}}_{1,3,4}$ which is written as a quadratic form in $m_{i}$.

In Step **S6**, using the Matlab program in [23] with ${\hat{E}}_{1,3,4}$ and ${\hat{C}}_{3,4,1}$ as the input, we find an SOS representation for ${\hat{E}}_{1,3,4}$. Thus, using Theorem 1, $C_{1}\left(3,4\right)$ is strictly proved.

## 4. Proof of C_3_(3,n) for n = 2, 3, 4 under the Log-Concave Condition

In this section, we use the procedure in Section 2.2 to prove $C_{3}\left(3,n\right)$ for n=2,3,4 under the log-concave condition. The detailed lengthy formulas can be seen in [21].

### 4.1. Compute E_3,3,n_

In **step 1**, we compute $E_{3,3,n}$ in (8) and (20):

(29) $$\begin{array}{c} \frac{1}{2}\frac{d^{2}}{dt^{2}}\left(\frac{∥\nabla p_{t}{∥}^{2}}{p_{t}}\right)-\frac{1}{n^{3}}E{\left(\frac{∥\nabla p_{t}{∥}^{2}-p_{t}\nabla^{2}p_{t}}{p_{t}^{2}}\right)}^{3}\\ \overset{\left(2\right)}{=}\int_{R^{n}}\frac{E_{3,3,n}}{p_{t}^{5}}dx_{t} \end{array}$$

where

$$E_{3,3,n}=\sum_{a=1}^{n}\sum_{b=1}^{n}\sum_{c=1}^{n}E_{3,a,b,c}$$

and

$$\begin{array}{cc} E_{3,a,b,c}\;\;\;\; & =\frac{p_{t}^{4}}{4}\frac{\partial^{3}p_{t}}{\partial x_{a,t}\partial^{2}x_{c,t}}\frac{\partial^{3}p_{t}}{\partial x_{a,t}\partial^{2}x_{b,t}}-\frac{p_{t}^{3}}{4}\frac{\partial p_{t}}{\partial x_{a,t}}\frac{\partial^{3}p_{t}}{\partial x_{a,t}\partial^{2}x_{b,t}}\frac{\partial^{2}p_{t}}{\partial^{2}x_{c,t}}\\ \; & +\frac{p_{t}^{4}}{4}\frac{\partial p_{t}}{\partial x_{a,t}}\frac{\partial^{5}p_{t}}{\partial x_{a,t}\partial^{2}x_{b,t}\partial^{2}x_{c,t}}-\frac{p_{t}^{3}}{4}\frac{\partial p_{t}}{\partial x_{a,t}}\frac{\partial^{3}p_{t}}{\partial x_{a,t}\partial^{2}x_{c,t}}\frac{\partial^{2}p_{t}}{\partial^{2}x_{b,t}}\\ \; & +\frac{p_{t}^{2}}{4}{\left(\frac{\partial p_{t}}{\partial x_{a,t}}\right)}^{2}\frac{\partial^{2}p_{t}}{\partial^{2}x_{b,t}}\frac{\partial^{2}p_{t}}{\partial^{2}x_{c,t}}-\frac{p_{t}^{3}}{8}{\left(\frac{\partial p_{t}}{\partial x_{a,t}}\right)}^{2}\frac{\partial^{4}p_{t}}{\partial^{2}x_{b,t}\partial^{2}x_{c,t}}\\ \; & -\frac{1}{n^{3}}\left[{\left(\frac{\partial p_{t}}{\partial x_{a,t}}\right)}^{2}-p_{t}\left(\frac{\partial^{2}p_{t}}{\partial^{2}x_{a,t}}\right)\right]\left[{\left(\frac{\partial p_{t}}{\partial x_{b,t}}\right)}^{2}-p_{t}\left(\frac{\partial^{2}p_{t}}{\partial^{2}x_{b,t}}\right)\right]\left[{\left(\frac{\partial p_{t}}{\partial x_{c,t}}\right)}^{2}-p_{t}\left(\frac{\partial^{2}p_{t}}{\partial^{2}x_{c,t}}\right)\right]. \end{array}$$

### 4.2. Compute the Third-Order Log-Concave Constraints

In **step 2**, we obtain the third-order log-concave constraints.

From Lemma 3, we can compute the third-order log-concave constraints:

(30) $$C_{3,2}=\left\{R_{1}=-{▵}_{1,1}Q_{1},R_{2}=-{▵}_{1,2}Q_{2},R_{3}={▵}_{2,1}Q_{3}\right\},$$

where $Q_{1},Q_{2}\in {\mathrm{Span}}_{R}\left(M_{4,4}\right)$ and $Q_{3}\in {\mathrm{Span}}_{R}\left(M_{2,2}\right)$. Note that $C_{3,2}$ does not contain all the log-concave constraints in Lemma 3. The constraints $C_{3,2}$ are enough for our purpose in this paper.

For n>2, we give certain log-concave constraints in a special form, which are needed in the proof procedure in Section 4.3. Let

$$\begin{array}{c} \nabla_{1}p_{t}=\left(\frac{\partial p_{t}}{\partial x_{a,t}},\frac{\partial p_{t}}{\partial x_{b,t}},\frac{\partial p_{t}}{\partial x_{c,t}}\right),\\ L_{1}\left(p_{t}\right)≜p_{t}H_{1}\left(p_{t}\right)-\nabla_{1}^{T}p_{t}\nabla_{1}p_{t}, \end{array}$$

where

$$H_{1}\left(p_{t}\right)=\left[\begin{array}{ccc} \frac{\partial^{2}p_{t}}{\partial^{2}x_{a,t}} & \frac{\partial^{2}p_{t}}{\partial x_{a,t}\partial x_{b,t}} & \frac{\partial^{2}p_{t}}{\partial x_{a,t}\partial x_{c,t}}\\ \frac{\partial^{2}p_{t}}{\partial x_{a,t}\partial x_{b,t}} & \frac{\partial^{2}p_{t}}{\partial^{2}x_{b,t}} & \frac{\partial^{2}p_{t}}{\partial x_{b,t}\partial x_{c,t}}\\ \frac{\partial^{2}p_{t}}{\partial x_{a,t}\partial x_{c,t}} & \frac{\partial^{2}p_{t}}{\partial x_{b,t}\partial x_{c,t}} & \frac{\partial^{2}p_{t}}{\partial^{2}x_{c,t}} \end{array}\right],$$

and ${▵}_{k,l}^{\prime },l=1,\ldots ,L_{k}$ the *k*th-order principle minors of $L_{1}\left(p_{t}\right)$. Let $M_{k}^{\prime }$ be the set of all monomials in $V_{a,b,c}$ (defined in (27)) which have a degree of *k* and a total order of *k*. We have

(31) $$\begin{array}{cc} C_{3,n}=\;\;\;\;\;\; & \left\{-{▵}_{1,1}^{\prime }Q_{1,1},-{▵}_{1,2}^{\prime }Q_{1,2},-{▵}_{1,3}^{\prime }Q_{1,3},{▵}_{2,1}^{\prime }Q_{2,1},{▵}_{2,2}^{\prime }Q_{2,2},{▵}_{2,3}^{\prime }Q_{2,3},-{▵}_{3,1}^{\prime }Q_{3,1}\right\} \end{array}$$

where $Q_{1,i}\in {\mathrm{Span}}_{R}\left(M_{4}^{\prime }\right)$, $Q_{2,j}\in {\mathrm{Span}}_{R}\left(M_{2}^{\prime }\right)$, and $Q_{3,1}\in R$.

### 4.3. Proof of C_3_(3,2)

The proof follows Procedure 2 with $E_{3,3,2}$ given in (29) and the constraints in (28) and (30) as the input.

Steps **S1**–**S3** are the same with the proof of the case $C_{1}\left(3,2\right)$.

In Step **S4**, we obtain $\hat{C}\left(3,2\right)$ which contains three quadratic-form constraints.

In Step **S5**, by eliminating the non-quadratic monomials in $E_{3,3,2}$ using $C_{3,2,2}$ to obtain a quadratic form in $m_{i}$ and then simplifying the quadratic form using $C_{3,2,1}$, we have

$$\begin{array}{cc} {\hat{E}}_{3,3,2}\;\;\;\;\; & =\frac{31}{40}m_{14}^{2}-\frac{147}{8}m_{13}^{2}-\frac{5}{2}m_{7}m_{10}+\frac{15}{4}m_{8}^{2}-\frac{25}{8}m_{9}^{2}\\ \; & -\frac{31}{16}m_{9}m_{11}+\frac{207}{8}m_{9}m_{13}-\frac{5}{8}m_{10}^{2}+\frac{1}{2}m_{1}^{2}\\ \; & -\frac{5}{4}m_{1}m_{5}+\frac{31}{40}m_{11}^{2}+\frac{31}{8}m_{12}^{2}+\frac{1}{2}m_{4}^{2}-\frac{5}{2}m_{4}m_{6}\\ \; & -\frac{5}{4}m_{4}m_{7}+\frac{3}{2}m_{3}^{2}-\frac{15}{4}m_{7}^{2}-\frac{5}{4}m_{4}m_{10}\\ \; & -\frac{5}{8}m_{5}^{2}+\frac{15}{8}m_{6}^{2}+\frac{3}{2}m_{2}^{2}-\frac{15}{4}m_{2}m_{6}. \end{array}$$

In Step **S6**, using the Matlab software in Appendix A [21] with ${\hat{E}}_{3,3,2}$, ${\hat{C}}_{3,2,1}$ and ${\hat{C}}_{3,2}$ as the input, we find an SOS representation for ${\hat{E}}_{3,3,2}$. Thus, $C_{3}\left(3,2\right)$ is proved under the log-concave condition. The Maple program for proving $C_{3}\left(3,2\right)$ can be found at https://github.com/cmyuanmmrc/codeforepi/ (accessed on 15 July 2020).

**Remark** **4.** *We fail to prove $C_{2}\left(3,2\right)$ even under the log-concave condition using the above procedure. Specifically, we cannot find an SOS representation for ${\hat{E}}_{2,3,2}$ in Step* **S6** *. Since the SDP algorithm is not complete for problem (21), we cannot say that an SOS representation does not exist for ${\hat{E}}_{2,3,2}$. The Maple program for $C_{2}\left(3,2\right)$ can be found at https://github.com/cmyuanmmrc/codeforepi/ (accessed on 15 July 2020).*

### 4.4. Proof of C_3_(3,3) and C_3_(3,4)

In this subsection, we prove $C_{3}\left(3,3\right),C_{3}\left(3,4\right)$. Motivated by symmetric functions, for any function f(a,b,c), we have

(32) $$\begin{array}{c} \sum_{a,b,c=1}^{n}f\left(a,b,c\right)=\sum_{1\leq a<b<c}^{n}\left\{\frac{2}{\left(n-1)(n-2\right)}[f\left(a,a,a\right)\right.\\ \;\;\;\;+f\left(b,b,b\right)+f\left(c,c,c\right)]+\frac{1}{n-2}[f\left(a,a,b\right)+f\left(a,b,a\right)\\ \;\;\;\;+f(b,a,a)+f(a,a,c)+f(a,c,a)+f(c,a,a)\\ \;\;\;\;+f(b,b,a)+f(b,a,b)+f(a,b,b)+f(b,b,c)\\ \;\;\;\;+f(b,c,b)+f(c,b,b)+f(c,c,a)+f(c,a,c)\\ \;\;+f(a,c,c)+f(c,c,b)+f(c,b,c)+f(b,c,c)]\\ \;\;\;\;+[f(a,b,c)+f(a,c,b)+f(b,a,c)+f(b,c,a)\\ \;\;\;\;+f(c,a,b)+f(c,b,a)]\}. \end{array}$$

From (29) and (32), we obtain

$$\begin{array}{c} E_{3,3,n}=\sum_{a=1}^{n}\sum_{b=1}^{n}\sum_{c=1}^{n}E_{3,a,b,c}=\sum_{1\leq a<b<c\leq n}^{n}J_{3,3,n}, \end{array}$$

where

(33) $$\begin{array}{cc} J_{3,3,n}\;\;\;\; & =\frac{2}{\left(n-1)(n-2\right)}\left[E_{3,a,a,a}+E_{3,b,b,b}+E_{3,c,c,c}\right]\\ \; & +\frac{1}{n-2}[E_{3,a,a,b}+E_{3,a,b,a}+E_{3,b,a,a}+E_{3,a,a,c}\\ \; & +E_{3,a,c,a}+E_{3,c,a,a}+E_{3,b,b,a}+E_{3,b,a,b}+E_{3,a,b,b}\\ \; & +E_{3,b,b,c}+E_{3,b,c,b}+E_{3,c,b,b}+E_{3,c,c,a}+E_{3,c,a,c}\\ \; & +E_{3,a,c,c}+E_{3,c,c,b}+E_{3,c,b,c}+E_{3,b,c,c}]\\ \; & +[E_{3,a,b,c}+E_{3,a,c,b}+E_{3,b,a,c}+E_{3,b,c,a}\\ \; & +E_{3,c,a,b}+E_{3,c,b,a}] \end{array}$$

From (33), if we prove $J_{3,3,n}\geq 0$, then $E_{3,3,n}\geq 0$. It is clear that $J_{3,3,n}$ has many fewer terms than $E_{3,3,n}$.

In $J_{3,3,n}$ given in (33) and the constraints in (28) and (31), we may consider $\frac{\partial }{\partial x_{a,t}}$, $\frac{\partial }{\partial x_{b,t}}$, and $\frac{\partial }{\partial x_{c,t}}$ as the differential operators without giving concrete values to a,b, and *c*.

First, we prove $C_{3}\left(3,3\right)$ using Procedure 2 with $J_{3,3,3}$ given in (33) and the constraints in (28) and (31) as the input.

In Step **S1**, the new variables are $M_{3}^{\prime }=\left\{m_{i},i=1,\ldots ,38\right\}$, which is the set of all the monomials in $R[V_{a,b,c}]$ with a degree of 3 and a total order of 3.

In Step **S2**, the constraints are: $C_{3,n}=\left\{R_{i,a,b,c}^{\left(3\right)}\;:\;i=1,\ldots ,955\right\}$, $N_{1}=955$. We obtain $C_{3,n,1}$ and $C_{3,n,2}$, which contain 350 and 328 constraints, respectively.

In Step **S3**, there exist 189 intrinsic constraints. In total, ${\hat{C}}_{3,n,1}$ contains 539 constraints. Using R-Gaussian elimination in ${\mathrm{Span}}_{R}\left({\hat{C}}_{3,n,1}\right)$ shows that 512 of these 539 constraints are linearly independent, thus $N_{3}=512$.

In Step **S4**, we obtain ${\hat{C}}_{3,n}$ from $C_{3,n}$ which contains six constraints.

In Step **S5**, eliminating the non-quadratic monomials in $J_{3,3,3}$ using $C_{3,n,2}$ and then simplifying the expression using $C_{3,n,1}$, we obtain ${\hat{J}}_{3,3,3}$, which is written as a quadratic form in $m_{i}$.

In Step **S6**, using the Matlab software in Appendix A [21] with ${\hat{J}}_{3,3,3}$, ${\hat{C}}_{3,n,1}$ and ${\hat{C}}_{3,n}$ as the input, we find an SOS representation for ${\hat{J}}_{3,3,3}$. Thus, using Theorem 1, $C_{3}\left(3,3\right)$ is strictly proved. The Maple program used to prove $C_{3}\left(3,3\right)$ can be found at https://github.com/cmyuanmmrc/codeforepi/ (accessed on 15 July 2020).

To prove $C_{3}\left(3,4\right)$, we just need to replace the input from $J_{3,3,3}$ with $J_{3,3,4}$ in Step **S5** in the above procedure. In the same way, $C_{3}\left(3,4\right)$ can be strictly proved. The Maple program used to prove $C_{3}\left(3,4\right)$ can be found at https://github.com/cmyuanmmrc/codeforepi/ (accessed on 15 July 2020).

## 5. Proof of C_3_(4,2)

In this section, we use the procedure in Section 2.2 to prove $C_{3}\left(4,2\right)$ under the log-concave condition.

In **step 1**, we compute $E_{3,4,n}$ in (8) and (20):

(34) $$\begin{array}{c} \frac{1}{2}\frac{d^{3}}{dt^{3}}\left(\frac{∥\nabla p_{t}{∥}^{2}}{p_{t}}\right)-\frac{3}{n^{4}}E{\left(\frac{∥\nabla p_{t}{∥}^{2}-p_{t}\nabla^{2}p_{t}}{p_{t}^{2}}\right)}^{4}\\ \overset{\left(2\right)}{=}\int_{R^{n}}\frac{E_{3,4,n}}{p_{t}^{7}}dx_{t}, \end{array}$$

where $E_{3,4,n}=\sum_{a=1}^{n}\sum_{b=1}^{n}\sum_{c=1}^{n}\sum_{d=1}^{n}E_{4,a,b,c,d}$. For brevity, we omit the concrete expression of $E_{4,a,b,c,d}$.

In **step 2**, based on Lemma 2, we obtain 589 fourth-order constraints:

(35) $$\begin{array}{c} C_{4,2}=\left\{R_{i}^{\left(2\right)}\;:\;i=1,\ldots ,589\right\}\subset R\left[P_{4,2}\right]\;\mathrm{and}\;N_{1}=589. \end{array}$$

Using Lemma 3, we obtain three fourth-order log-concave constraints:

$$C_{4,2}=\left\{-{▵}_{1,1}Q_{1,1},-{▵}_{1,2}Q_{1,2},{▵}_{2,1}Q_{2,1}\right\}$$

where $Q_{1,1},Q_{1,2}\in {\mathrm{Span}}_{R}\left(M_{6,2}\right)$ and $Q_{2,1}\in {\mathrm{Span}}_{R}\left(M_{4,2}\right)$.

In **step 3**, we use Procedure 2 to compute the SOS representations (13) and (14) with $E_{3,4,n}$, $C_{4,2}$, and $C_{4,2}$ as the input.

In Step **S1**, the new variables are $M_{4,2}=\left\{m_{i},i=1,\ldots ,33\right\}$, which is the set of all monomials in $R[P_{4,2}]$ with a degree of 4 and a total order of 4, and which is listed in the lexicographical monomial order.

In Step **S2**, using Gaussian elimination for $C_{4,2}=\left\{R_{i}^{\left(2\right)}\;:\;i=1,\ldots ,589\right\}$, we obtain $C_{4,2,1}$ and $C_{4,2,2}$, which contain 266 and 182 constraints, respectively.

In Step **S3**, there exist 182 intrinsic constraints. Thus, ${\hat{C}}_{4,2,1}$ contains 448 constraints. Using R-Gaussian elimination in ${\mathrm{Span}}_{R}\left({\hat{C}}_{4,2,1}\right)$ shows that 417 of these 448 constraints are linearly independent, so $N_{3}=417$.

In Step **S4**, we obtain $\hat{C}\left(4,2\right)$, which contain three log-concave constraints, so $N_{2}=3$.

In Step **S5**, by eliminating the non-quadratic monomials in $E_{3,4,2}$ using $C_{4,2,2}$ to obtain a quadratic form in $m_{i}$ and then simplifying the quadratic form using $C_{4,2,1}$, we obtain ${\hat{E}}_{3,4,2}$ which is written as a quadratic form in $m_{i}$.

In Step **S6**, using the Matlab software in Appendix A of [21] with ${\hat{E}}_{3,4,2}$, ${\hat{C}}_{4,2,1}$ and $\hat{C}\left(4,2\right)$ as the input, we find an SOS representation for ${\hat{E}}_{3,4,2}$. Thus, using Theorem 1, $C_{3}\left(4,2\right)$ is strictly proved under the log-concave condition. The Maple program used to prove $C_{3}\left(4,2\right)$ can be found at https://github.com/cmyuanmmrc/codeforepi/ (accessed on 15 July 2020).

## 6. Conclusions

In this paper, three conjectures $C_{l}\left(m,n\right)$ for l=1,2,3 concerning the lower bound for the derivatives of $H(X_{t})$ are considered. We propose a general procedure to prove inequities similar to $C_{l}\left(m,n\right)$. We first consider one of the conjectures of McKean $C_{1}\left(m,n\right):{\left(-1\right)}^{m+1}\left(d^{m}/dt^{m}\right)H\left(X_{t}\right)\geq 0$ in the multivariate case, and prove $C_{1}\left(3,2\right)$, $C_{1}\left(3,3\right)$ and $C_{1}\left(3,4\right)$. This conjecture is also mentioned in Villani’s paper [14], and is named the super-H theorem. Motivated by $C_{2}\left(m,n\right)$, we further propose the following weaker conjecture $C_{3}\left(m,n\right):{\left(-1\right)}^{m+1}\left(d^{m}/dt^{m}\right)H\left(X_{t}\right)$$\geq {\left(-1\right)}^{m+1}\frac{1}{n}\left(d^{m}/dt^{m}\right)H\left(X_{Gt}\right)$. Using our procedure, we prove $C_{3}\left(3,2\right),C_{3}\left(3,3\right),C_{3}\left(3,4\right)$ and $C_{3}\left(4,2\right)$ under the log-concave condition.

In the univariate case (n=1), $C_{1}\left(3,1\right)$ and $C_{1}\left(4,1\right)$ were proved [16] and $C_{1}\left(5,1\right)$ cannot be proved with the SDP approach (In this paper, when we say $C_{s}\left(m,n\right)$ cannot be proved with the SDP approach, we mean that the software in Appendix A of [21] terminates and gives a negative answer for problem (21)) [18,22]. $C_{2}\left(3,1\right)$, $C_{2}\left(4,1\right)$, and $C_{2}\left(5,1\right)$ were proved under the log-concave condition [18]. We try to prove $C_{2}\left(6,1\right)$ under the log-concave condition. However, due to the accuracy of the SDP software, we cannot find an explicit SOS representation. In the multivariate case, $C_{1}\left(3,2\right)$, $C_{1}\left(3,3\right)$, and $C_{1}\left(3,4\right)$ were proved and $C_{1}\left(4,2\right)$ cannot be proved with the SDP approach [22]. For $C_{1}\left(3,n\right),n>4$, the corresponding SDP problem is too large for the Matlab software in Appendix A [23]. In this paper, $C_{3}\left(3,2\right)$, $C_{3}\left(3,3\right)$, $C_{3}\left(3,4\right)$, and $C_{3}\left(4,2\right)$ were proved under the log-concave condition, and $C_{2}\left(3,2\right)$, $C_{2}\left(3,3\right)$, $C_{2}\left(3,4\right)$, and $C_{2}\left(4,2\right)$ cannot be proved with the SDP approach under the log-concave condition. For $C_{3}\left(3,n\right),n>4$ and $C_{3}\left(4,n\right),n>2$, the corresponding SDP problems are too large for the Matlab software in Appendix A [21].

In order to use the SDP approach to prove more difficult problems, two kinds of improvements are needed. First, it is easy to see that the size of $E_{s}\left(m,n\right)$ and the numbers of the constraints increase exponentially as *m* and *n* become larger. Thus, we need to find certain rules which could be used to simplify the computation to solve problems such as $C_{1}\left(3,n\right)\left(n>4\right)$ and $C_{3}\left(3,n\right)\left(n>4\right)$ under the log-concave condition. Second, in many cases, such as $C_{1}\left(5,1\right)$ and $C_{2}\left(3,2\right)$ under the log-concave constraint, the SDP software terminates and gives a negative answer. Since the SDP method is not complete for our problem, we do not know whether an SOS representation exists. We thus need a complete method to solve problem (13). Another problem is to find more constraints besides those used in this paper in order to increase the power of the approach.

## Figures and Tables

**Table 1:** Data in computing the SOS with symbolic computation and SDP.

	$C_{2}\left(3,1\right)$	$C_{1}\left(3,2\right)$	$C_{1}\left(3,3\right)$	$C_{1}\left(3,4\right)$	$C_{3}\left(3,2\right)$	$C_{3}\left(3,3\right)$	$C_{3}\left(3,4\right)$	$C_{3}\left(4,2\right)$
Vars	3	14	38	80	14	38	38	33
$N_{1}$	6	63	512	1966	63	512	512	417
$N_{2}$	0	0	0	0	0	6	6	3

## Data Availability

Not applicable.
